# Nutrient Intake and Digestibility, Performance, and Carcass Characteristics of Sheep Kept on Massai Grass Pasture and Subjected to Intermittent Supplementation

**DOI:** 10.3390/ani16071067

**Published:** 2026-04-01

**Authors:** Stela Antas Urbano, Alana Santos de Freitas, Naira Cristina Ribeiro Pimentel, Yasmin dos Santos Silva, Maria Alice de Lima Soares, Dorgival M. de Lima Júnior, João Virgínio Emerenciano Neto, Pedro Henrique Cavalcante Ribeiro, Adriano Henrique do Nascimento Rangel

**Affiliations:** 1 Academic Unit Specialized in Agrarian Sciences, Federal University of Rio Grande do Norte, Macaíba 59280-000, RN, Brazil; ala_fre18@outlook.com (A.S.d.F.); nairapimentel1@hotmail.com (N.C.R.P.); yasminsszoo@gmail.com (Y.d.S.S.); mariaalicesoares07@gmail.com (M.A.d.L.S.); joao.emerenciano@ufrn.br (J.V.E.N.); pedrohcrib@gmail.com (P.H.C.R.); adrianohrangel@yahoo.com.br (A.H.d.N.R.); 2 Department of Animal Sciences, Federal Rural University of the Semi-Arid, Mossoró 59625-900, RN, Brazil; juniorzootec@yahoo.com.br

**Keywords:** sheep meat, tissue composition, sheep nutrition, *Panicum maximum*, carcass yield

## Abstract

This study aimed to assess the effect of concentrate supplementation frequency on the nutrient intake, performance, carcass characteristics, and tissue composition of sheep for meat raised on *Panicum maximum* cv. Massai pasture. A group of 36 sheep of the Santa Inês breed (18 males and 18 females) with a mean initial weight of 17.0 ± 1.5 kg and mean age of 90 ± 10 days were assigned to three treatments: supplementation every 24 h, every 48 h, and every 72 h. The formulation comprised ground maize kernels (70%), soybean meal (25%), mineral mix (2.5%), urea (1.8%), table salt (0.5%), and ammonium sulfate (0.2%). The females reached a higher final weight than the males (*p* < 0.05). Dry matter intake did not differ among the treatment groups (*p* > 0.05), but the sheep supplemented every 72 h showed greater pasture and neutral detergent fiber (NDF) intake, while those supplemented every 24 h and 48 h consumed more concentrate and had higher total weight gain (7.19 and 7.10 × 5.68 kg) (*p* < 0.05). The same behavior was found for carcass and meat cut weights and for leg and carcass compacity indexes, but carcass and cut yields were not impacted by supplementation strategy. Supplementation on alternate days, i.e., every 48 h, maintains the performance and productive efficiency levels in sheep kept on Massai grass pasture compared to those supplemented daily, but providing intermittent supplementation with intervals above 48 h is not recommended as a nutritional strategy.

## Simple Summary

Reducing the age at slaughter of sheep for meat is a basic premise to improve meat quality and, therefore, increase the per capita consumption of that type of protein. Nutrition and nutritional management strategies that converge towards that objective are studied, and we hypothesize that giving an intermittent supply of concentrate supplements to sheep kept on pasture might maintain the nutrient intake and digestibility, performance, carcass characteristics, and tissue composition obtained in animals supplemented daily. Providing supplements in intervals above 48 h is not recommended, but supplementation on alternate days is a nutritional management alternative that does not decrease productive indexes and may be implemented in sheep meat production systems that use pasture as a basis of nutrition.

## 1. Introduction

Semi-arid regions carry a natural vocation for sheep farming, likely due to the climate characteristics of those regions and the tolerance and adaptability of the species, which is more hardy and tolerant to harsh climates, and drives producers to invest in short-cycle systems focused on meat production. However, production chain fragilities, stemming from the lack of application of nutrition, management, and genetic technologies, result in the frequent commercialization of carcasses that did not originate in specialized meat production systems [1], which results in supplying the market with products lacking the quality standard demanded by modern consumers. Therefore, it is speculated that reducing the age at slaughter leads to improvements in the quality of the meat that reaches final consumers and tends to increase the *per capita* consumption of this product in Brazil, thus supporting sheep farming for meat and contributing to the consolidation of the industry [2,3].

Providing concentrate supplements has been pointed to as a vital technique to maintain the sustainability of production systems based on pasture [4], which experience variations in the amount and quality of feed, with direct impacts on their productivity. However, combining supplements and forage seems challenging, and success depends on optimizing ruminal fermentation efficiency, seeking to increase the supply of metabolizable energy and microbial protein synthesis, which will invariably improve animal performance and favor reducing age at slaughter [5].

Given that supplying concentrate itself generates costs through the purchase of ingredients, transportation, and distribution of the concentrate in pastures, it is possible that supplying the supplement at a lower frequency results in a more favorable cost–benefit ratio since the method allows for optimizing and rationalizing labor, fuel, and machinery associated with the supplementation process [6,7]. Animals could possibly compensate for the intake of forage/supplement [8] on days on which the concentrate is or is not supplied and therefore maintain similar performance or show little variation when compared with those supplemented daily, as pointed out by Urbano et al. [8], who found, in the same experimental area, a mean daily weight gain (DMG) of 0.06 kg when providing supplementation on alternate days, with no significant difference in performance.

Thus, it is hypothesized that decreasing supplementation frequency does not compromise animal performance or nutrient intake in sheep kept on Massai grass, and the present study aimed to assess the effect of concentrate supplementation frequency on nutrient intake and digestibility, performance, carcass characteristics, and tissue composition of sheep raised for meat kept on *Panicum maximum* cv. Massai grass pasture.

## 2. Materials and Methods

The research proposal was submitted for evaluation by the Ethics Commission on Animal Use of UFRN and was approved under certificate 191.043/2019. The study was conducted in accordance with the recommendations by the National Council of Animal Experimentation Control (*Conselho Nacional de Controle da Experimentação Animal*—CONCEA).

The trial was carried out at the experimental area of the Academic Unit Specialized in Agricultural Sciences of the Federal University of Rio Grande do Norte, located in Macaíba, RN, Brazil. The climate in the region is characterized as dry sub-humid [9], with an average monthly rainfall of 230 mm during the experimental period.

The trial lasted for 120 days (25 February 2020 to 25 June 2020), the first 20 of which were used for the animals to adapt to the management and experimental facilities, and the other 100, for data collection. The total area of the experimental *Panicum maximum* cv. Massai pasture was 1.44 ha (divided into three paddocks of 0.48 ha), delimited by a screen fence and equipped with a drinking trough. Concentrate supplementation was provided in a managed barn with 36 individual stalls, all equipped with feeding and drinking troughs. A group of 36 sheep of the Santa Inês breed (18 males and 18 females), with a mean ± SD initial weight of 17.0 ± 1.5 kg and mean ± SD age of 90 ± 10 days, was used.

Throughout the experimental period, the animals were kept on pasture from 8 a.m. to 4 p.m. and fed concentrate supplementation in individual pens in the afternoon, where they stayed overnight. The pastures were managed under continuous stocking with a variable stocking rate as a function of animal weight. The mean forage mass during the experimental period was 3367 kg dry matter/ha at 62% leaf blades and 38% stems. The average stocking rate during the experimental period was 17.33 AU (Animal Unit—30 kg)/ha, and the average forage availability was 6.47 kg DM/kg LW.

The different supplementation frequencies assessed were treatment 24—daily supplementation (every 24 h); treatment 48—supplementation on alternate days (every 48 h); and treatment 72—supplementation every three days (every 72 h). The animals in treatments 48 and 72 were managed the same way as those in treatment 24, being taken to the stalls daily at 4 p.m., but with no supplementation provided on some days, according to the treatment plan. All sheep were provided with mean supplementation equivalent to 1.0% live weight (LW) concentrate/day, with the amount being adjusted according to the weight of each animal. Animals supplemented every 48 or 72 h received, respectively, double or triple the amount on the day of feeding.

The supplement was formulated following the recommendations by the NRC [10] to meet the maintenance requirements of animals with a 20 kg body weight, allowing for gains of 100 g/day. The formulation comprised ground maize kernels (70%), soybean meal (25%), mineral mix (2.5%), urea (1.8%), table salt (0.5%), and ammonium sulfate (0.2%).

The forage and ingredients of the concentrate supplement were pre-dried in a forced air oven and then ground in a grinding mill, with 1 mm sieves for analysis of contents of dry matter (DM; method 934.01), mineral matter (MM; method 930.05), crude protein (CP; method 968.06), ether extract (EE; method 920.39), and organic matter (OM = 100 − MM) following the methodology described by the AOAC [11]. Neutral detergent fiber (NDF) and acid detergent fiber (ADF) contents were determined according to the methodology described by Detmann et al. [12], using heat-stable α-amylase and corrected for ash.

In order to follow the weight evolution, adjust supplement amounts, and calculate DMG, the animals were weighed at the start of the experimental period and every 14 days after solid fasting for 16 h, always in the morning before being released to pasture. Total weight gain (TWG) was obtained from the difference between final body weight (FW) and initial body weight (IW), TWG = (FW − IW), while mean daily gain (MDG) was obtained from the ratio between TWG and the number of days of the data collection period (100 days).

Voluntary dry matter intake was estimated using the combination of an external indicator (LIPE^®^, Research Products Simões Saliba P2S2, Florestal—Minas Gerais, Brazil) and an internal one (NDFi). Fecal dry matter production was estimated by the external indicator, provided orally (capsules) at 2.5 g/day at 8 a.m. for seven days (two days of adaptation and five of collection). Feces were collected directly from the rectal ampulla once a day at different times (8 a.m., 10 a.m., 12 p.m., 2 p.m., and 4 p.m.) over the five days of collection, with a compound sample formed for each animal at the end of the collection period. Those samples were later dried in a forced air oven (55 °C), ground in a grinding mill with a 1 mm sieve, and submitted for reading in a spectrophotometer (410 nm) for determination of LIPE^®^ concentrations according to the methodology described by Myers [13].

The indigestible neutral detergent fiber (NDFi) fractions of the diets and feces were determined by in situ incubation of samples of the ingredients, Massai grass, and feces in the rumen of a sheep for 288 h, following the methodology described by Valente et al. [14]. The samples were placed in TNT bags (100 g/cm^2^; 5 cm × 5 cm) containing 0.5 g of sample and incubated in the rumen in triplicate. During incubation, the donor sheep had access to Massai grass pasture and received a daily amount of concentrate (same formulation as the experimental animals) equivalent to 1% of their body weight. After that period, the material remaining from incubation was submitted to extraction with neutral detergent for quantification of NDFi contents.

After the 100 days of collection, the males were submitted to water fasting for 16 h and then stunned by brain concussion [15] and, after unconsciousness was verified, they were bled, skinned, eviscerated, and had their heads and feet removed. A potentiometer equipped with an insertion probe (Testo^®^, model 205) was used to measure carcass pH and temperature immediately after slaughter and 24 h *post mortem* at the semimembranosus muscle. The carcasses were weighed to obtain the hot carcass weight (HCW) and were then placed in a cold chamber (4 ± 1 °C) hanging from the common calcaneal tendon for 24 h.

The gastrointestinal tract was weighed full and empty (the contents were removed manually and the tract was rinsed with running water) to determine empty body weight (EBW) and biological or true yield [TY(%) = HCW/EBW × 100]. After refrigeration, a tape measure was used to measure the internal and leg lengths, in addition to croup width, which were used to calculate the compacity indexes of the leg (quotient between croup width and leg length) and of the carcass (quotient between cold carcass weight and internal carcass length). After the measurements, the carcasses were weighed again to obtain the cold carcass weight (CCW) and to calculate the hot carcass yield (HCY(%) = HCW/BWS × 100) and commercial yield (CY(%) = CCW/BWS × 100), where BWS is body weight at slaughter.

The carcasses were sectioned lengthwise, and the half-carcasses were weighed, with the left-side half-carcasses being divided into six anatomic regions to yield six commercial meat cuts (neck, shoulder, leg, loin, ribs, and breast), which were weighed to calculate their respective yields.

The left-side hind leg of each animal was vacuum sealed and stored at −18 °C for assessment of tissue composition according to the methodology described by Brown and Williams [16]. Tissue weights and yields, as well as the muscle/bone and muscle/fat ratios, were obtained. The leg muscularity index (LMI) was calculated according to Purchas et al. [17] using the following formula: LMI = √(W5M/FL)/FL, where W5M is the weight of the five main muscles surrounding the femur (g) and FL is the femur length.

A completely randomized factorial experimental design in a 2 × 3 factorial arrangement was employed, with two sexes, three supplementation frequencies, and six repetitions per treatment for a total of 36 plots. Analysis of variance (ANOVA) was performed for sex, supplementation frequencies, and their interactions, and, when needed, the averages were compared by Tukey’s test at 5% probability in the software SAS 9.4 using the following mathematical model:

Y_ijk_ = µ + S_i_ + F_j_ + SF_ij_ + β(X_ijk_ − X) + ɛ_ijk_,

where

Y_ijk_ = observed value;

µ = overall average of the experiment;

S_i_ = fixed effect of sex (i = male or female);

F_j_ = fixed effect of supplementation frequency (j = 1, 2, 3);

SF_ij_ = effect of the interaction sex × supplementation frequency;

β(X_jk_ − X) = effect of co-variable (initial body weight of animal)

ɛ_jk_ = random experimental error.

Since the females were not slaughtered, their carcass data were assessed only for the influence of supplementation frequency using the following model:

Y_jk_ = µ + F_j_ + β(X_jk_ − X) + ɛ_jk_,

where

Y_jk_ = observed value;

µ = overall average of the experiment;

F_j_ = fixed effect of supplementation frequency (j = 1, 2, 3);

β(X_jk_ − X) = effect of co-variable (initial body weight of animal);

ɛ_jk_ = random experimental error.

## 3. Results

The chemical composition of the diet components is shown in Table 1.

No effect of the sex × supplementation frequency interaction was found for any of the variables analyzed (*p* > 0.05). Supplementation frequency impacted pasture consumption (PASC), concentrate intake, neutral detergent fiber intake (NDFC), dry matter digestibility (DMD), roughage/concentrate ratio (R:C), and feed conversion (FC) (Table 2). Sex had an effect only on FC, which was also impacted by supplementation frequency.

The animals supplemented at the different frequencies assessed showed no significant difference in dry matter consumption (DMC); however, higher PASC and NDFC values were found for the animals supplemented every 72 h. Concentrate consumption (CONCC) also statistically differed between the supplementation frequencies employed, being the lowest for the animals supplemented every 72 h, as shown in Table 2. Dry matter digestibility was similar and higher for the animals supplemented every 24 h or 48 h when compared to those in the 72 h group. No effect on NDF digestibility was observed. The R:C ratio was the highest for the animals supplemented every 72 h. The highest FC values were found for males and animals supplemented every 72 h (Table 2).

The animals supplemented every 24 h and 48 h showed higher DMG, TWG, and FW (Table 3). Higher FW was observed for females, with no significant differences found for the other parameters.

Animals supplemented every 24 h and 48 h had the highest EBW, HCW, CCW, and leg and carcass compacity indexes (*p* < 0.05), but no effect of supplementation strategy was found on carcass pH, temperature, or yields (*p* > 0.05) (Table 4).

Except for the loin, lower weights were found for all commercial meat cuts when the animals were supplemented every 72 h. Otherwise, cut yields were not impacted by nutritional strategy (Table 5).

The tissue composition of the leg (Table 6) was impacted by supplementation frequency, except for the bone weight and muscle/fat ratio (*p* > 0.05). Overall, the weights of tissue components that translate into meat were higher for the animals supplemented every 24 h and 48 h. This is supported by the behavior observed for LMI, which was lower for the legs of animals supplemented every 72 h. A higher bone proportion was found in the animals supplemented every 72 h, with no effect on the other tissue component yields assessed.

## 4. Discussion

As supplementation intervals increased from 24 to 48 to 72 h, concentrate intake decreased while pasture intake increased, indicating a partial substitution of concentrate for forage without net change in total DMI (g/day and %LW). The substitutive effect impacted the R:C ratio, which favored roughage in the animals supplemented every 72 h, and in NDFC, which showed a similar behavior. Therefore, these behaviors explain, through the likely reduction in energy intake in the animals supplemented at lower frequency, the results found for the carcass performance and characteristics and tissue composition of the sheep assessed in this experiment.

The lower DMD is also associated with the substitutive effect found for the animals supplemented every 72 h given the lower energy supply to the ruminal environment, which may have reduced microbial protein synthesis, thus compromising the efficiency of fiber degradation by rumen microorganisms [18].

Intermittent concentrate supplies may have led to alterations in the intake behavior and ruminal environment of the animals supplemented every 72 h, since they were fed, all at once, an amount of supplement equivalent to three days of supply for the animals supplemented daily. That may have induced the animals to prioritize forage intake during grazing as an adaptative strategy [8]. Higher pasture and NDF intakes were also observed by Paula et al. [6] in ruminants grazing Marandu grass who were supplemented three times a week when compared with those supplemented daily.

Although ruminal pH was not measured in this trial, it is also speculated that, for the animals supplemented every 72 h, the accumulated concentrate supply may have contributed to a decrease in ruminal pH on the days of supplementation, triggering a post-prandial experience that may have been negatively associated with supplement intake, which then modulated intake behavior and depressed concentrate intake. Indeed, Morais et al. [8] associated the constancy of dry matter intake and digestibility in ruminants supplemented intermittently with the stability of the ruminal environment. Therefore, it is understood that the changes in R:C ratios found between treatments impacted the energy density of the diets, hence compromising the FC and performance of sheep in the finishing phase [19].

The results obtained in the present research for final weight are higher than those reported by Pereira et al. [20], who used a similar supplement as the one in this study for male Santa Inês sheep fed Massai grass and achieved a mean final weight of 19.1 kg. Paula et al. [6] found lower values when using supplementation at different frequencies for animals on pasture (0.050 kg for daily frequency and 0.060 kg for alternate frequency). In contrast, Urbano et al. [2] reported higher final body weight results when using different supplementation frequencies for sheep finished on Massai pasture in the same experimental area.

Besides the changes in energy intake, it is also noteworthy that, in physiological terms, the intermittent supplementation strategy is based on the ability of ruminants to conserve nitrogen for long periods, possibly through changes in the permeability of the gastrointestinal tract, thus maintaining nitrogen supply to the rumen ecosystem between the supplementation periods [21]. Hence, since Massai grass is rich in NDF, which is difficult to digest [22] and requires intense activity of the fibrolytic flora, whose multiplication is stimulated by ruminal nitrogen, it is inferred that the ruminal nitrogen pool of the animals supplemented every 24 h or 48 h was kept within the levels required by the rumen microbiota, with no depreciation of ability to digest fiber. That contrasts with what was observed in the 72 h treatment, which explains the similarities between the 24 h and 48 h treatments.

The better feed conversion of females can be explained by the similar growth rate of young animals of either sex, since young males do not experience significant influences from androgen hormones. That contributes to females being able to achieve similar and even higher performance gains, including due to their precocity in fat deposition and carcass finishing [23,24], which is confirmed by their higher final weight (Table 4).

Since feed consumption is the natural pathway and the only way to intake the nutrients that will be converted into tissues to be deposited in the empty body [25], the difference in CONC found explains the effects observed for EBW, HCW, CCW, commercial cuts, LCI, CCI, and LMI, as the lower concentrate intake limits the energy intake and availability for greater development of muscle and fat tissues in the carcasses [26,27]. HCY, CCY, and BY were not impacted by the supplementation strategy, which supports the argument that what took place was actually lower tissue deposition in the empty body and not interference of the gut on absolute values.

No influence of supplementation frequencies was found on commercial cut yields, which can be attributed to the homogeneity of the animals used in the research (age and breed), thus reducing the phenotypical variability of the carcasses and contributing for the body regions to maintain similar proportions between the treatments. That result supports the law of anatomical harmony described by Boccard & Dumont [28], according to which carcasses with similar weights and fat contents have a proportional distribution of cuts.

The lower values found for the absolute weights of tissue components in the animals supplemented every 72 h are also due to the lower concentrate intake observed in that group, which reduces energy intake and can impair protein deposition in the empty body, compromising, among other things, muscle development. [29]. Silva et al. [30] also reported that lambs submitted to higher protein and energy supplementation levels on *Brachiaria* pastures or in confinement had higher muscle proportions and lower bone proportions, leading to better muscle/bone ratios.

Despite the differences in tissue composition found, particularly for subcutaneous fat, no alteration was found in carcass cooling or in post-mortem reactions, which is confirmed by the maintenance of carcass temperature and pH values within the standards described by Cezar and Sousa (2007) [31] for sheep carcasses.

## 5. Conclusions

Sheep kept on Massai grass pasture and supplemented daily or on alternate days showed similar performance, nutrient intake and digestibility, and carcass and commercial cut weights, but showed higher performance than those supplemented every 72 h, which makes providing intermittent supplementation with intervals above 48 h not recommended as a nutritional strategy.

## Figures and Tables

**Table 1:** Chemical composition of the experimental diet components.

Components (g/kg Dry Matter)	Ingredients		
	Massai Grass	Soybean Meal	Maize
Dry matter ^1^	366.9	922.2	909.8
Mineral matter	56.1	71.8	13.1
Organic matter	943.9	928.2	986.9
Crude protein	15.4	452.6	79.9
Neutral detergent fiber	781.4	144.8	119.0
Acid detergent fiber	447.8	94.4	42.3

^1^ g/kg natural matter.

**Table 2:** Pasture, concentrate, dry matter, neutral detergent fiber intake, digestibility coefficients, roughage/concentrate ratio, and feed conversion of male and female sheep kept on Massai grass pasture supplemented at different frequencies.

Variables	Sex		Frequency			SEM	*p*-Value		
	Male	Female	24 h	48 h	72 h		Sex	Frequency	S × F
PASC (g/day)	300.5	288.5	268.6 ^b^	293.9 ^ab^	321.1 ^a^	40.74	0.3830	0.0136	0.6050
CONCC (g/day)	186.2	191.4	208.1 ^a^	211.1 ^a^	147.3 ^b^	24.25	0.5249	<0.0001	0.9712
DMC (g/day)	486.8	480.1	476.7	505.1	468.4	49.08	0.6797	0.1762	0.7649
DMC (%LW)	2.367	2.294	2.258	2.338	2.396	0.32	0.5081	0.5849	0.6157
NDFC (g/day)	248.9	239.8	225.3 ^b^	245.4 ^ab^	262.5 ^a^	32.21	0.4032	0.0288	0.6146
DMD %	49.09	47.11	49.16 ^a^	50.54 ^a^	44.60 ^b^	4.32	0.1819	0.0057	0.7268
NDFD %	34.49	31.23	29.64	34.24	34.70	6.60	0.1486	0.1332	0.5094
R:C	68:32	60:40	56:44 ^b^	58:42 ^b^	68:32 ^a^	4.29	0.2506	<0.0001	0.6909
FC	11.90 ^A^	8.26 ^B^	8.90 ^ab^	7.95 ^b^	13.38 ^a^	5.27	0.0474	0.0391	0.3232

PASC = pasture consumption; CONCC = concentrate consumption; DMC = dry matter consumption; NDFC = neutral detergent fiber consumption; DMD = dry matter digestibility; NDFD = neutral detergent fiber digestibility; R:C = roughage/concentrate ratio; FC = feed conversion; LW = live weight. Means followed by different capital or small letters in the same row differ at 5% significance according to Tukey’s test.

**Table 3:** Performance of male and female sheep kept on Massai grass pasture supplemented at different frequencies.

Variables	Sex		Frequency			SEM	*p*-Value		
	Male	Female	24 h	48 h	72 h		Sex	Frequency	S × F
FW (kg)	23.63 ^B^	25.57 ^A^	26.00 ^a^	24.81 ^ab^	22.99 ^b^	2.44	0.0241	0.0177	0.8951
TWG (kg)	6.41	6.90	7.19 ^a^	7.10 ^a^	5.68 ^b^	1.35	0.2880	0.0172	0.7042
MDG (kg)	0.064	0.069	0.071 ^a^	0.071 ^a^	0.057 ^b^	0.01	0.2873	0.0163	0.7024

FW = final weight; TWG = total weight gain; MDG = mean daily weight gain; S × F = sex × frequency. Means followed by different capital or small letters in the same row differ at 5% significance according to Tukey’s test.

**Table 4:** Carcass characteristics of sheep kept on Massai grass pasture subjected to intermittent supplementation.

Variables	Supplementation Frequency				
	24 h	48 h	72 h	SEM	*p*-Value
HCW (kg)	9.07 ^a^	9.58 ^a^	7.85 ^b^	0.163	0.0021
CCW (kg)	8.72 ^a^	9.11 ^a^	7.54 ^b^	0.150	0.0023
EBW (kg)	17.60 ^a^	18.11 ^a^	15.50 ^b^	0.181	0.0001
HCY (%)	39.13	39.70	35.90	0.377	0.0998
CCY (%)	37.61	37.70	34.49	0.656	0.1116
BY (%)	51.33	52.76	50.33	0.963	0.1828
pH 0 h	6.70	6.89	6.90	0.032	0.0853
pH 24 h	5.44	5.46	5.43	0.034	0.0795
Temperature (0 h)	34.38	35.35	34.74	0.396	0.0734
Temperature (24 h)	6.97	6.95	7.40	0.231	0.1232
LCI	0.541 ^a^	0.551 ^a^	0.517 ^b^	0.008	0.0005
CCI	0.154 ^a^	0.160 ^a^	0.135 ^b^	0.005	0.0005

HCW = hot carcass weight; CCW = cold carcass weight; EBW = empty body weight; HCY = hot carcass yield; CCY = cold carcass yield; BY = biological yield; LCI = leg compacity index; CCI = carcass compacity index. Means with different letters on the same row differ at 1% significance according to Tukey’s test.

**Table 5:** Weights and yields of meat cuts of sheep kept on Massai grass pasture subjected to intermittent supplementation.

Variables	Supplementation Frequency				
	24 h	48 h	72 h	SEM	*p*-Value
Weight (g)					
Shoulder	878 ^a^	896 ^a^	748 ^b^	19.161	0.0136
Neck	442 ^ab^	471 ^a^	391 ^b^	9.385	0.0120
Ribs	740 ^a^	764 ^a^	609 ^b^	11.671	0.0002
Breast	413 ^ab^	435 ^a^	338 ^b^	12.887	0.0207
Loin	417	422	373	9.665	0.1153
Leg	1513 ^a^	1562 ^a^	1269 ^b^	33.686	0.0069
Yield (%)					
Shoulder	20.04	19.68	20.29	0.227	0.5620
Neck	10.01	10.38	10.44	0.158	0.4909
Ribs	16.85	16.88	16.26	0.216	0.4423
Breast	9.34	9.50	9.01	0.219	0.6542
Loin	9.36	9.28	9.90	0.135	0.1571
Leg	34.41	34.28	34.10	0.308	0.9186

Means with different letters on the same row differ at 5% significance according to Tukey’s test.

**Table 6:** Leg tissue composition of sheep kept on Massai grass pasture subjected to intermittent supplementation.

Variables	Supplementation Frequency				
	24 h	48 h	72 h	SEM	*p*-Value
Weight (g)					
Muscle	985 ^ab^	1026 ^a^	833 ^b^	24.534	0.0157
Subcutaneous fat	63.5 ^ab^	80.3 ^a^	43.2 ^b^	4.241	0.0106
Total fat	98.2 ^ab^	115.7 ^a^	65.6 ^b^	5.782	0.0104
Bone	310	342	312	6.074	0.0845
Ratio (g)					
Muscle/bone	3.15 ^a^	2.98 ^ab^	2.64 ^b^	0.058	0.0087
Muscle/fat	10.14	9.65	13.21	0.698	0.1145
Yield (%)					
Muscle	67.20	67.04	65.96	0.524	0.5886
Subcutaneous fat	4.57	5.17	3.55	0.284	0.0987
Total fat	6.98	7.45	5.27	0.406	0.1062
Bone	21.76 ^b^	22.71 ^b^	25.33 ^a^	0.338	0.0020
Index (kg/cm)					
Leg muscularity	0.343 ^a^	0.346 ^a^	0.312 ^b^	0.003	0.0044

Means with different letters on the same row differ at 5% significance according to Tukey’s test.

## Data Availability

The original contributions presented in this study are included in the article. Further inquiries can be directed to the corresponding author.
